# Effect of fluoride slow‐release glass devices on salivary and gingival crevicular fluid levels of fluoride: A pilot study

**DOI:** 10.1002/cre2.227

**Published:** 2019-07-28

**Authors:** Chrysoula Tatsi, Kyriacos Jack Toumba

**Affiliations:** ^1^ Department of Paediatric Dentistry, Leeds School of Dentistry University of Leeds Leeds LU UK

**Keywords:** fluoride, fluoride electrode, fluoride slow‐release glass devices, gingival crevicular fluid, ion chromatography

## Abstract

**Objectives:**

To estimate the effect of fluoride slow‐release glass devices on the levels of fluoride in a pooled sample of human gingival crevicular fluid and in human saliva.

**Materials and Methods:**

Ten healthy adult volunteers wore fluoride slow‐release glass devices for 3 months in a longitudinal experimental clinical pilot study. Whole unstimulated human saliva and gingival crevicular fluid were collected using paper points at baseline, after 2 weeks and at 3 months and analysed for their fluoride levels using ion chromatography and fluoride electrode.

**Results:**

No adverse effects were reported, and the Löe Plaque and Gingival Index remained low (0.22). The saliva determination of fluoride using the fluoride electrode showed an increase after 3 months from 0.02 ± 0.04 ppm to 0.06 ± 0.12 ppm, whereas the ion chromatography showed an increase from 0.15 ± 0.10 ppm to 0.44 ± 0.36 ppm. The fluoride levels in a pooled sample of gingival crevicular fluid from four intraoral sites were determined using the ion chromatography, and the results showed that after 3 months, the fluoride levels were still low (0.71 ± 0.34 ppb) similar to those at baseline (0.74 ± 0.31 ppb).

**Conclusions:**

The fluoride concentration in a pooled sample of gingival crevicular fluid was reported to be low with a range from 0.46 to 0.75 ppb and was not changed by placement of fluoride slow‐release glass devices. The fluoride concentration in unstimulated human saliva showed an increase after 3 months when the fluoride slow‐release glass devices were attached when determined with both the fluoride electrode (from .02 ± 0.04 ppm to 0.06 ± 0.12 ppm) and ion chromatography (from 0.15 ± 0.10 ppm to 0.44 ± 0.36 ppm).

## 1 INTRODUCTION

Caries is a disease involving a dynamic process of demineralisation and remineralisation of the dental enamel hydroxyapatite emerging when the control over this balance is lost in favour of demineralisation. Fluoride (F) as a free ion has a key role with its topical action when present in the plaque–enamel interface being of most importance in preventing caries (Fejerskov, Thylstrup, & Larsen, 2009). Amongst various F delivery methods that have been tested, the fluoride slow‐release glass devices (FSRGDs) have been shown to effectively reduce caries incidence in children over a 2‐year period (Toumba & Curzon, 2005).

Dental caries affects both the occlusal and the smooth surfaces, which are in close proximity with the gingivae, forming a crevice that can be less than a 1 mm up to 2–3 mm depth in gingivae free of inflammation. The crevice contains the gingival crevicular fluid (GCF), first recognised over 100 years ago (Black, 1899) and fully described in 1958 (Brill & Krasse, 2009). It bathes the cervical area of the tooth; however, its role in the caries process is not fully understood.

Textbooks state the GCF is “unlikely to be an important source of F for plaque and/or plaque fluid” (Fejerskov, Ekstrand, & Burt, 1996) based on the findings of two dog‐animal studies (MacFadyen, Hilditch, Stephen, Horton, & Campbell, 1979; Whitford, Pashley, & Pearson, 1981) investigating F in the GCF in correlation to blood plasma, concluding that this fluid is a possible source of F in the cervical area of the tooth. In humans, the concentration has been correlated to the blood plasma levels, reported to range from 1 μM (0.019 ppm F) in an area with 1.0 ppm F in the drinking water (Fejerskov et al., 1996) to 0.7–2.4 μM (0.0133–0.0456 ppm F) in fluoride‐free areas (Johansen, Taves, & Olsen, 1979).

The FSRGDs was chosen because it has been shown to increase levels of fluoride in saliva (Toumba & Curzon, 2005) and it has no effect on blood plasma levels (Curzon & Toumba, 2004).

More recent work investigated F clearance using the fluoride electrode (FE) in fluid in the interdental area after tooth brushing and rinsing. Samples were obtained from alternative sites with custom‐made filter paper points following the methodology of a study to monitor F release from toothpicks and dental flosses (Kashani, Birkhed, & Petersson, 1998). Paper points were transferred to 50‐ml liquid (total ionic strength adjustment buffer [TISAB] and distilled water 1:10) in a 0.5‐ml Eppendorf tube. The mean F concentration (mM) was plotted versus time (min), and the area under the curve was calculated. The baseline measurements were not displayed numerically on the graph, but there was an increase of up to 0.87 mM (16.53 ppm F) 5 min after tooth brushing that reached 0.08 mM (1.52 ppm F) after 180 min (Sjögren, Birkhed, Rangmar, & Reinhold, 1996).

The same methodology was used in a similar randomised crossover study, investigating the duration of rinsing and volume of water. F concentration in a log scale (mmol) was plotted against time (min). Even though no actual numerical results are reported, the authors state that: “there was a tendency towards a numerically higher F concentration in interdental fluid sampled after the rinsing routines using less water”(Sjögren & Melin, 2001).

For determination of F in saliva, the FE was used, a widely accepted method in dental research (Andreadis, Toumba, & Curzon, 2006; Curzon & Toumba, 2004; Sjögren et al., 1996; Toumba & Curzon, 2005). However, its detection limit (0.1 ppm) does not allow accuracy for low levels of F, and because it determines the whole F in the sample being unable to differentiate how much of it is in its active form, that is, as a free ion, it has been suggested that this may be misleading (McCabe, Carrick, & Sidhu, 2002).

IC is considered the method of choice for analytical determination of free ions, with a detection limit of 1 ppb (part per billion) (Fritz, 2004). Its low detection limit 0.001 ppm = 1 ppb (McCabe et al., 2002) enables determination of very low concentrations of free F ions, which is the active form of the F in terms of caries prevention. IC separates ions through ion exchange in a separating column that is intolerant to organic species (e.g., proteins); therefore, a saliva sample may have serious consequences for the separating column. (McCabe et al., 2002) In dental research, it has been used in in vitro studies to measure F in distilled–deionized water (al Ibrahim, Tahmassebi, & Toumba, 2010; Itota et al., 2004; Itota, Carrick, Yoshiyama, & McCabe, 2004; Kapetania, Toumba, Watson, & Robinson, 2005; McCabe et al., 2002) and in human saliva in clinical studies. The aim of this paper was to investigate the F concentration of human GCF and to perform measurements using IC. The objectives of the study were

1. to measure F in a pooled sample of human GCF using IC;
2. to measure F in whole unstimulated saliva using FE and IC;
3. to assess any changes in the F levels of saliva and GCF in patients having FSRGDs over a period of 3 months.

## 2 MATERIALS AND METHODS

The Leeds West Research Ethics Committee gave a favourable opinion for the study (reference number 05/Q1205/122). A signed informed consent was obtained from all volunteers. The work described has been carried out in accordance with the Code of Ethics of the World Medical Association (Declaration of Helsinki) for experiments involving humans. A convenient sample of 10 healthy adult dentate volunteers with a minimum of 0.25 ml/min of salivary flow rate were recruited and were asked to use F‐free toothpaste (The Boots Company PLC, Nottingham, UK) for 1 week before their sampling visit. Participants were not asked to refrain from specific food(s)/drink(s) before their sampling appointment. The first permanent molars were tested for Löe Plaque and Gingival Index and were found to be free of caries and restorations on the smooth surfaces. Gingival health was part of the inclusion criteria as participants had a Löe Plaque and Gingival Index <1.

**Figure 1:**
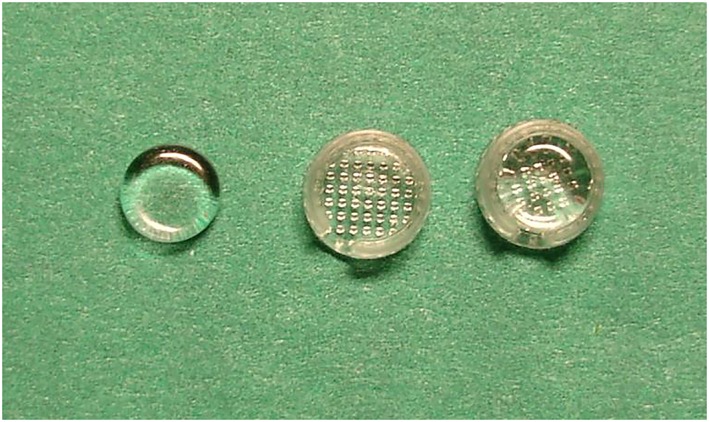
Photo of a slow‐release fluoride glass device with plastic holder

**Figure 2:**
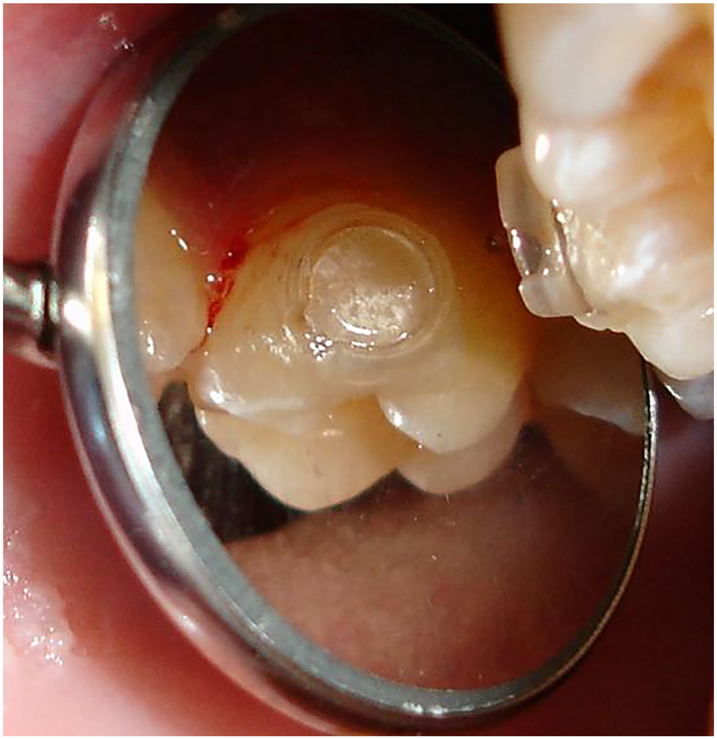
In situ slow‐release fluoride glass device attached on buccal surface of maxillary first permanent molar

FSRGDs (13.3% F, Ultradent Products Inc, 505 W 10200 S, South Jordan, UT 84095, USA) were attached on the buccal surfaces of the maxillary first permanent molars as this has been found to be the optimum place to achieve highest salivary F levels, as seen in Figure 1 (Toumba & Curzon, 2005). Teeth were etched with 37% orthophosphoric acid for 30 s and were washed and dried for a further 30 s. Light‐cured bonding agent (Prime & Bond, 3 M) was applied followed by light‐curing composite resin (Spectrum Universal) and placement of the devices. Any excess composite resin was removed before light curing, and the occlusion was checked as seen in Figure 2.

Absorbent paper points (ISO 030 colour coded 4%, Dentsply, Maillefer, France) were prepared with 2 M hydrochloric acid. The paper points were left in the acid for 24 hr in a glass vial and washed and left in distilled water that was changed daily until neutral pH 7.0 checked with a calibrated pH electrode (Orion 520A). They were left to dry in a Petri dish in an incubator at 37°C for 24–48 hr. Paper points in groups of four were placed in microcentrifuge tubes and were weighed before and immediately after sampling of GCF by using a five‐figure electronic balance (Ohaus Analytical Plus) in order to allow estimation of the F concentration.

GCF was sampled from the buccal crevices of the lower first permanent molars and the palatal surfaces of the upper first permanent molars with the paper point left in situ for 5 min per side, gently inserted into the crevice until resistance was felt(Brill, 1962). Saliva/plaque from the area was removed with the help of a swab, whereas cheek retractors, dry guards, cotton rolls, and a saliva aspirator were used to prevent saliva contamination. Deionized water was used as storage medium, and samples were kept at 4°C in a refrigerator until analysis.

Whole unstimulated saliva was collected for 2 min by asking volunteers to drool and spit in sterile plastic tubes and was stored at −12°C in a freezer until analysis.

GCF and whole unstimulated saliva were sampled immediately before placement of FSRGDs and after 2 weeks and 3 months. Samples were collected by the same investigator (C. T.) who was trained to perform the analysis.

Biochemical analysis of saliva requires removal of all proteins that may interfere with the assays. The preparation method used has a significant 93.3% protein removal (Benzo et al., 2002). Accordingly, 250 μl of each saliva sample was placed in a microcentrifuge tube, and 25 μl of NaOH (2 M) and 75 μl of H_2_O were added and shaken for a few seconds. Then, 500 μl of acetonitrile was added, and the resulting mixture was shaken for 10 s in a vortex machine before centrifuged for 12 min at 20, 200 rcf speed, at 26°C in an Eppendorf Centrifuge 5417R machine (Eppendorf‐Netheler‐Hinz‐GmbH, Hamburg, Germany). Finally, 200 μl of the supernatant solution was placed in the vial of the ion chromatograph ready to be injected by the autosampler.

The 761 Compact Ion Chromatography (Metrohm Ion Analysis, Metrohm LTD, Herisau, Switzerland) was used for analysis, and the Hamilton PRP × 110S 7 μm 250 × 4.1 mm column with the Metrohm RP Guard column was selected for analysing the fluoride in saliva and GCF with a carbonate eluent (NaHCO_3_ 1.7 mmol/L and Na_2_CO_3_ 1.8 mmol/L) at 0.5 ml/min of flow rate. All the functions were controlled by using 761 PC Software 2.2 (Metrohm Ion Analysis, Metrohm LTD, Herisau, Switzerland). Each chromatogram of each sample was opened on the screen, and calculation of the F concentration in ppm/ppb was obtained after selection and measurement of the peak area. The software program calibrated the samples with the standard solutions in order to allow measurements of the F concentration.

Analysis of saliva using the FE requires use of TISAB, which was added to the saliva samples at 1:1 dilution.

### 2.1 Statistical analysis of data

Descriptive statistics and confidence intervals were used for the data using a computer software package (SPSS, version 13).

### 2.2 Results

**Figure 3:**
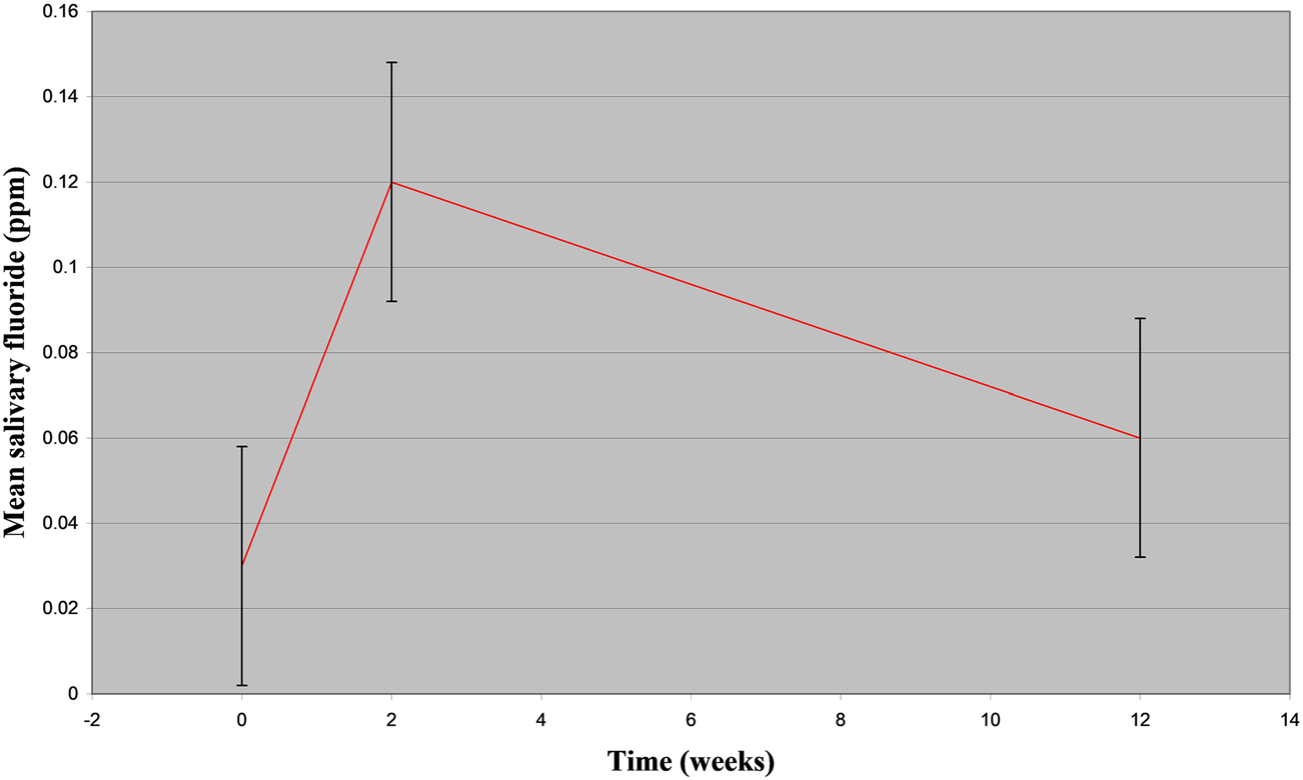
Mean (±*SD*) fluoride (ppm) in saliva measured with the fluoride electrode at baseline, 2 weeks and 3 months in 10 volunteers with fluoride slow‐release glass devices

**Figure 4:**
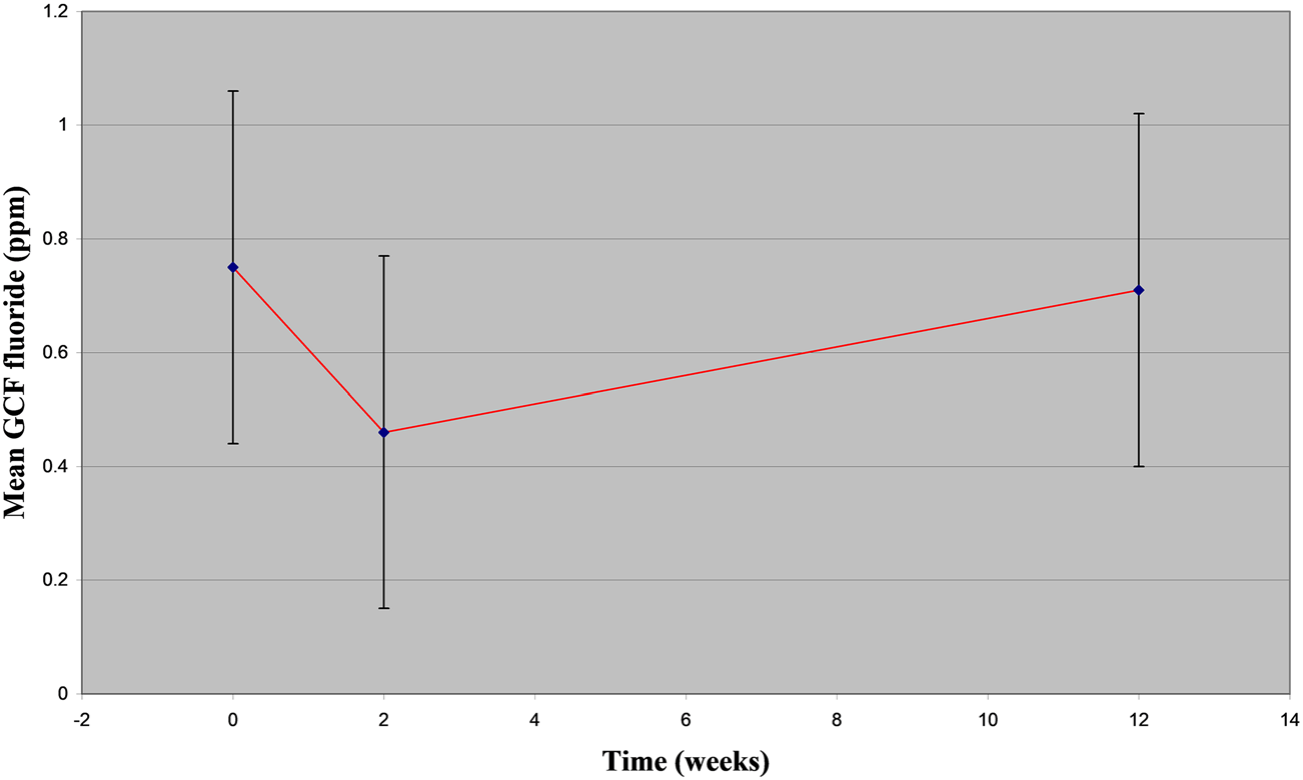
Mean (±*SD*) gingival crevicular fluid fluoride measured with ion chromatography at baseline, 2 weeks and 3 months, in 10 volunteers using the fluoride slow‐release glass devices

In total, 20 FSRGDs were attached in 10 volunteers. No systemic adverse effects were reported. However, four volunteers lost one device each within the first week of the trial mainly when biting on hard food, but they were replaced within 2 days. Retention rate was 80% (16/20) for the 3‐month period of this study. The mean Löe Plaque and Gingival Index was 0.22, indicating plaque recognised only by running a probe across the tooth surface and no gingival bleeding on pressure (Löe & Silness, 2009). F concentration in deionized water measured by IC was estimated to be 0.0043 ± 0.0049 ppm. Results for F concentration (in ppm) in saliva measured by the FE and IC are shown in Table 1 and Figure 3. Results for F concentration in GCF (in ppb) measured by IC are seen in Table 2 and Figure 4.

**Table 1:** F (ppm) in whole unstimulated saliva in *n* = 10 volunteers with fluoride slow‐release glass devices

F (ppm)				
	Fluoride electrode		Ion chromatography	
Time (weeks)	Mean ± *SD*	Range	Mean ± *SD*	Range
0	0.03 ± 0.04	0.01–0.14	0.15 ± 0.11	0.04–0.39
2	0.12 ± 0.16	0.01–0.58	0.34 ± 0.28	0.17–1.01
12	0.06 ± 0.13	0.01–0.42	0.44 ± 0.38	0.09–1.21

**Table 2:** Fluoride (ppb) in gingival crevicular fluid saliva in *n* = 10 volunteers with fluoride slow‐release glass devices

Fluoride (ppb)		
Time (weeks)	Mean ± *SD*	Range
0	0.75 ± 0.31	0.22–1.19
2	0.46 ± 0.44	0.01–1.40
12	0.71 ± 0.35	0.22–1.14

## 3 DISCUSSION

The aim of this pilot study was to investigate and assess changes in F concentration in a pooled sample of human GCF and whole unstimulated saliva in volunteers who had FSRGDs for a period of 3 months. Gingival inflammation increases production of GCF; therefore, it was recorded using the Löe Plaque and Gingival Index. In order to eliminate any influence from other F sources, the volunteers were asked to refrain from using any F supplements and/or F toothpaste for 1 week before each sampling appointment but were not asked to refrain from specific food(s)/drink(s) as it would be impractical to determine which types should be avoided and for how long. Sampling took place immediately before placement of FSRGDs and after 2 weeks and 3 months to allow comparison with results from previous studies. All volunteers lived in Leeds, UK, where the F in the drinking water is below the optimal level (<0.1 ppm). Retention of the FSRGDs (80%) was comparable with a previous study that reported 86% retention (13/15) during a period of 6 months (Andreadis et al., 2006).

### 3.1 Fluoride in saliva using the FE

Initially, there was an increase after 2 weeks from 0.02 to 0.12 ppm, followed by a decrease after 3 months to a level of 0.06 ppm, which was still greater than baseline. Baseline findings are in accordance with other studies investigating the unstimulated salivary F in nonfluoridated areas with reported levels of 0.026 ppm (Bruun & Thylstrup, 1984) or in fluoridated areas with reported levels of 0.01–0.03 ppm (Dawes & Weatherell, 2016). A comparison with studies investigating FSRGDs showed similar results with an increase from 0.009 to 0.031 ppm after 7 months (Curzon & Toumba, 2004). However, they appear to be much lower compared with another study with adults, which showed an increase after 6 months from 0.02 to 0.15 ppm (Andreadis et al., 2006). The majority of the reported results appear to be below the detection limit of the FE (0.1 ppm). Another possible explanation for these differences is the fact that different sampling methods were used for the collection of saliva. It has been shown that the presence of 0.01–0.04 ppm F in saliva can effectively inhibit demineralisation and enhance remineralisation of enamel lesions (Leverett, Adair, & Shield, 1987). FSRGDs can increase salivary F to such levels, and they have the advantage of being a constant intraoral source of F with no need for patients compliance, which makes them particularly useful for people in high caries‐risk groups.

### 3.2 Ion chromatography

IC in its modern form was introduced in 1975 (Small, Stevens, & Bauman, 2002) and has been used for analysis of ions in environmental samples, chemical/petrochemical industries, water applications, food and beverage applications, (Jackson, 2000) and body fluids (Benzo et al., 2002). In a study investigating blood plasma, it was reported that the separation columns original characteristics did not change; however, they concluded: “scarce information has been given about column integrity after this type of analysis”. (Abudiak, Robinson, Duggal, Strafford, & Toumba, 2012) The same methodology for chemical preparation of samples (Benzo et al., 2002) has been used to analyse F in human saliva (Abudiak et al., 2012; Kapetania et al., 2005). Few studies in dental research have used IC mainly in vitro, testing dental materials using distilled/deionized water, (Itota, Carrick, Rusby, et al., 2004; Itota, Carrick, Yoshiyama, et al., 2004; McCabe et al., 2002) but to our knowledge, no studies have attempted to use IC to analyse fluoride in GCF.

### 3.3 Fluoride in saliva using IC

In this study, the chemical preparation of the saliva sample was identical to previous studies (Abudiak et al., 2012; Benzo et al., 2002). The retention time of the F peak was not consistent, and in many cases, there were competing peaks from other ions appearing very close to the F peak; hence, the peak of interest was not identifiable. The concentration of F in saliva could not be determined using the eluent proposed for blood serum (Benzo et al., 2002) and the ones used for human saliva (Abudiak et al., 2012; Kapetania et al., 2005). Different eluents and combinations were used, namely, NaOH at different concentrations (12, 6, and 4 mM) and NaHCO_3_ with Na_2_CO_3_ at different concentrations (NaHCO_3_ 1.7 and 1.5 mmol/L and Na_2_CO_3_ 1.8 and 1.7 mmol/L). Different flow rates were also used for the sample to pass through the separation column from 1.5 to 0.5 ml/min, but the F peak was not clearly identifiable and, therefore, the concentration could not be determined. The F findings in the GCF led us to use paper points for sampling saliva. The same steps were followed as for the GCF and a clear and consistent F peak appeared at 9.3 min. A carbonate eluent was used (NaHCO_3_ 1.7 mmol/L and Na_2_CO_3_ 1.8 mmol/L) at a flow rate of 0.5 ml/min, the same as for the GCF sample but different to previous studies that used either 6‐mM NaOH or 12‐mM NaOH (Abudiak et al., 2012; Kapetania et al., 2005). The salivary F concentration in this study increased from 0.15 ± 0.10 ppm at baseline to 0.33 ± 0.26 ppm after 2 weeks and finally to 0.44 ± 0.36 ppm at 3 months. These findings were higher at baseline but lower after 3 months with baseline levels of 0.014 ± 0.02 ppm, whereas after 1 month, levels increased to 0.62 ± 1.00 ppm (Kapetania et al., 2005). The present studys findings appeared to be higher compared with the results after 7 days (0.057 ± 0.019 ppm F) and after 21 days (0.047 ± 0.028 ppm F) in a similar study (Abudiak et al., 2012). Because the chemical preparation of the samples was identical, the only possible explanation for the differences apart from the fact that the samples were not obtained at identical time points was the use of a different eluent in the IC.

### 3.4 Comparison between IC and FE for fluoride in saliva

There were difference when comparing the IC and the FE, with the IC reporting higher levels with the exception of measurement at 2 weeks. Even though readings from the IC were above the detection limit of the FE (0.1 ppm), still the measurements given by the FE were below its own detection limit. Further investigation is needed to explain these differences between the two methods of F analysis.

### 3.5 Fluoride in GCF

In this study, GCF was evaluated as a pooled sample from four different quadrants in the mouth due to its low volume estimated to be 0.06 μl in cases of gingival health (Lamster, Vogel, Hartley, DeGeorge, & Gordon, 1985; Persson & Page, 1990). It was decided to sample four sites for 5 min at each site in order to obtain an adequate amount of GCF but minimising extensive sampling periods. The sites chosen were the first permanent molars as it has been reported that there was a difference in the amount of GCF from anterior teeth (0.24–0.43 μl/tooth), whereas in molars, the amount of GCF has been estimated to range from 0.43 to 0.56 μl/tooth when the gingival index is <1. (Challacombe, 1980) The paper points were immediately weighed after sampling to minimise evaporation loss and determine the amount of GCF.

The mean F concentration in the pooled GCF samples was 0.747 ± 0.31 ppm F at baseline, at 2 weeks 0.459 ± 0.44 ppm F, and at 3 months 0.713 ± 0.34 ppm F measured with IC. There was a decrease after 2 weeks, but after 3 months, the F concentration was at the same level as baseline. Possible explanation for the fact that baseline F in the pooled GCF samples was higher compared with levels at 2 weeks and 3 months may be either not adherence to research protocol asking volunteers to refrain from the use of any F products for a week prior to sampling. Previous studies (Sjögren et al., 1996; Sjögren & Melin, 2001) have investigated the F concentration of interdental fluid sampled for 10 min and collected by placing custom‐made paper points in the area of adjacent posterior teeth after brushing and rinsing with different techniques in order to estimate F clearance from the area. Two sites were sampled three times each at various time points. The paper points were left overnight at 4°C in a 50‐μl solution (TISAB and distilled water 1:10) in a 0.5‐ml Eppendorf tube, and the FE was used to measure the F concentration (Sjögren et al., 1996). Evaporation is a constant threat when the sample volume is so small, and it is enhanced by a prolonged sampling period, which also causes an increase in protein concentration. Prolonged sampling periods had also been reported to cause changes in the GCF protein concentration (Curtis, Griffiths, Price, Coulthurst, & Johnson, 1988) and evaporation loss. These levels reported from these studies were quite high and different from the present study findings. However, the sampling technique was different, and the method of determining F was also different; hence, it is difficult to make comparisons.

The results of this study show that the FSRGDs have no effect on the GCF levels of F, possibly because the GCF is a dilution of blood plasma, whereas the effect of the devices is predominantly intraorally and not systemically. It has been shown that swallowing an FSRGD does not cause any increase in blood plasma F concentration (Curzon & Toumba, 2004). Therefore, no effect should be expected from the devices systemically, whereas the GCF has been strongly related to plasma(MacFadyen et al., 1979; Whitford et al., 1981) and estimation of its F concentration was based on blood plasma findings, estimated to be 1 μM (0.019 ppm F) when living in a fluoridated area (Fejerskov et al., 1996). However, the present study findings showed a high F concentration of 0.75 ppm F at baseline, 0.46 ppm F at 2 weeks, and 0.71 ppm F at 3 months. The GCF sample was a pooled sample from four intraoral sites, and this may explain the high F concentration. Because blood plasma was not collected, we could not correlate the GCF findings to blood plasma findings.

The GCF is considered a dilution of blood plasma, and it has been extensively investigated in relation to periodontal diseases. However, its role, if any, in the caries process has not been thoroughly investigated, but it is not considered a source of F for the plaque fluid in the enamel–plaque interface where demineralisation and remineralisation of the enamel occurs. Two studies have investigated the F content in the GCF and blood plasma in dogs (MacFadyen et al., 1979; Whitford et al., 1981) and reported that the F changes in the GCF reflect those of plasma. One study reported similar F concentrations in plaque and plasma, plasma and saliva, GCF and plasma, and GCF and plaque for a period of 1 hr following intravenous injection of radionuclide F. (Kapetania et al., 2005) As a result, the F levels of GCF might change similarly to plasma levels when there is a change in F in drinking water. Second, their results suggested that the GCF is one source of plaque F even though they could not assess how much was available for transfer from GCF to plaque (MacFadyen et al., 1979). The other animal study (Whitford et al., 1981) where various concentrations of NaF were infused intravenously led to the conclusion that F in GCF was identical to plasma levels and even though quantitative studies are missing, it must be regarded as a source of F for the oral environment. Markers were also used to investigate evaporative water loss, which was reported to be a major source of error in studies of concentration of substances in the GCF.

In this study, we estimated the levels of F in a pooled sample of GCF using IC. Even though the concentrations were estimated to be high (0.46–0.75 ppm F), they were not influenced by FSRGDs whose effect is predominantly topical. This supports the fact that GCF is considered a dilution of plasma and its concentration is strongly related to plasma. The topical effect of the devices was shown in the salivary F concentration measured with the FE and IC, which increased during the 3‐month period of this study. To our knowledge, there are no previous studies investigating F concentration in human GCF; therefore, no direct comparisons can be made and further investigation is needed.

This experimental pilot study had a convenient small sample of 10 volunteers with no sample size calculation and no control group. These limitations do not allow any meaningful comparisons to be made and/or any clinical indications.

## 4 CONCLUSIONS

- The F concentration in a pooled sample of GCF from four intraoral sites was estimated using IC to range from 0.46 to 0.75 ppm F.
- The F concentration of the GCF did not change after 3 months (0.71 ± 0.34 ppm F) in adult volunteers who had the FSRGDs compared with baseline (0.75 ± 0.31 ppm F).
- The F concentration in saliva when measured with the FE showed an increase from 0.02 ± 0.04 ppm F at baseline to 0.06 ± 0.12 ppm F after 3 months in adult volunteers who had the FSRGDs.
- The F concentration in saliva when measured with the IC showed an increase from 0.15 ± 0.10 ppm F at baseline to 0.44 ± 0.36 ppm F after 3 months in adult volunteers who had the FSRGDs.
- The F measurements in the saliva measured with the FE and the IC were different with the IC showing a higher concentration.

### KEY FINDINGS

Stable F levels in gingival crevicular fluid using fluoride slow‐release glass devices.

## CONFLICT OF INTEREST

The authors would like to declare no conflicts of interest.
